# Preoperative Resilience, Self‐Efficacy, and Grit Are Associated With Postoperative Functional Outcomes in the Sports Medicine Patient: A Systematic Review

**DOI:** 10.1002/ars2.70031

**Published:** 2026-05-21

**Authors:** Claude J. Regis, Terence L. Thomas, Harrison S. Fellheimer, Molly Milano, Graham S. Goh, Fotios P. Tjoumakaris, Kevin B. Freedman

**Affiliations:** ^1^ Sidney Kimmel Medical College Thomas Jefferson University Philadelphia Pennsylvania U.S.A.; ^2^ Rothman Orthopaedic Institute at Thomas Jefferson University Philadelphia Pennsylvania U.S.A.; ^3^ Department of Orthopaedic Surgery Boston University Medical Center Boston Massachusetts U.S.A.

## Abstract

**Purpose:**

To assess the impact of personality traits on postoperative functional outcomes in patients undergoing common orthopaedic sports medicine surgeries.

**Methods:**

A systematic review of PubMed (MEDLINE), Scopus (EMBASE, MEDLINE, COMPENDEX), and Cochrane Central database from inception to October 1, 2024, was conducted. Primary articles that investigated the relationship between preoperative psychosocial characteristics (i.e., resilience, grit, self‐efficacy, catastrophizing, locus of control, optimism) and postoperative surgical outcomes after undergoing sports medicine/arthroscopy procedures (shoulder, hip, and knee arthroscopy) were included.

**Results:**

A total of 20 studies investigating the association between preoperative personality traits and postoperative functional outcomes were reviewed. Among the personality traits examined, resilience (9 studies), pain catastrophizing (7 studies), self‐efficacy (3 studies), grit (2 studies), locus of control (1 study), and optimism (1 study) were analyzed. High preoperative resilience was significantly correlated with improved postoperative outcomes in 5 of 9 studies, particularly in knee arthroscopy cohorts. Pain catastrophizing showed mixed results, with 3 of 7 studies indicating a significant correlation between low pain catastrophizing and better postoperative outcomes. All 3 studies examining self‐efficacy showed a positive relationship with postoperative outcomes. Higher preoperative grit exhibited improved postoperative outcomes among all included studies, whereas locus of control and optimism had no significant impact in the 1 study that met the inclusion criteria. Among the 3 studies that explored relationship between baseline personality traits and minimal clinically important difference in postoperative outcomes, no studies found a significant correlation.

**Conclusions:**

Resilience, self‐efficacy, and grit emerged as personality traits with potential associations with postoperative functional outcomes in orthopaedic sports medicine patients. The remaining personality traits of pain catastrophizing, locus of control, and optimism showed either mixed results or no correlation with functional outcomes. When assessed, no study found a correlation between preoperative traits and achievement of minimal clinically important difference in outcomes.

**Level of Evidence:**

Level IV, systematic review of Level II to IV studies.

## Graphical abstract

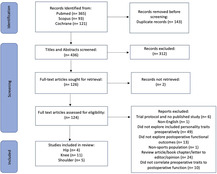

Studies have shown a relationship between mental health conditions such as anxiety, depression, and bipolar disorder and surgical outcomes.^1^, ^2^, ^3^ Alongside the aforementioned psychological diagnoses, a focus has been directed toward understanding the relationship between patient‐specific personality traits (e.g., resilience) and outcomes.^4^ In contrast to psychological disorders such as depression and anxiety, which can be treated with appropriate pharmacologic interventions, there is a range of distinct personality traits that are typically viewed as innate, untreated characteristics, shaped by dynamic epigenetic, psychosocial, and developmental factors in an individual's life.^5^ As an individual's perception of life stressors has a tremendous influence on one's ability to overcome challenges, a deeper understanding of the impact of personality traits related to resilience on overall postoperative functional recovery emerges as an important concept.^5^ Defining more clearly the relationship between personality traits and postoperative outcomes presents an opportunity to improve preoperative and postoperative patient optimization.

Despite growing interest in this topic, a comprehensive synthesis of literature evaluating preoperative personality traits as a predictor for postoperative functional outcomes is warranted. “Resilience” is often the primary term used to describe an individual's ability to perceive challenge and adapt to difficult circumstances; however, there are many surrogate terms that aid in measuring similar characteristics.^4^, ^5^ As previous reviews have solely explored the impact of resilience on arthroscopic surgical outcomes,^4^ a better understanding of the varying impact of a wider variety of personality traits on surgical results requires further evaluation.

The purpose of the present systematic review was to assess the impact of personality traits on postoperative functional outcomes in patients undergoing common orthopaedic sports medicine surgeries. The authors hypothesized that lower levels of resilience, self‐efficacy, grit, and optimism, along with high levels of pain catastrophizing, will be associated with poor functional outcomes in the postoperative period.

## METHODS

### Literature Search and Selection Criteria

A systematic review of PubMed (MEDLINE), Scopus (EMBASE, MEDLINE, COMPENDEX), and Cochrane Central database from inception to October 1, 2024 was conducted. A broad query was conducted across all 3 databases using search terms specific to the study population (orthopaedic sports medicine procedures including ligament reconstruction, knee arthroscopy, hip arthroscopy, elbow, and shoulder arthroscopy), intervention (resilience, grit, self‐efficacy, catastrophizing, locus of control), and outcome (functional, return to sport, patient‐reported outcome measures) (Table S1). The present study was registered on the International Prospective Register of Systematic Reviews (CRD42024550285).

All article titles and abstracts were initially reviewed independently by 3 authors (C.J.R., H.S.F., M.M.) for relevance. Investigators selected studies that aligned with the predefined criteria for inclusion and exclusion. Any disparities encountered during the screening of titles and abstracts were resolved by a fourth independent reviewer (T.L.T.). After article selection, all 4 authors conducted a detailed examination of the complete articles. Final determination of inclusion was based on meeting the following criteria: (1) population of patients who underwent orthopaedic sports medicine surgery (e.g., anterior cruciate ligament, posterior cruciate ligament, posterolateral corner, meniscus, ulnar collateral ligament, lateral collateral ligament surgery) or any arthroscopic (e.g., knee, hip, ankle, elbow, or shoulder) surgery; (2) quantitative assessment of preoperative personality traits including resilience, grit, self‐efficacy, catastrophizing, and locus of control; and (3) investigation of functional outcomes after surgery. Studies were excluded if the study population included nonhuman subjects or non–sports medicine patients. Additionally, if the paper was an expert opinion, commentary, letter to editor, systematic review, meta‐analysis, or textbook chapter, the study was excluded. Additionally, we excluded studies that only investigated psychiatric diagnoses without inclusion of personality traits, or studies that combined preoperative personality traits into a customized model as such data were not extractable, particularly if the model included traits that were not specified in the inclusion criteria.

### Data Evaluation and Statistical Analysis

Upon final inclusion, the following data were extracted from each study: year published, author, title, study design, patient follow‐up time, study period, number of patients, patient sex, number of procedures, patient demographics, confounding mental conditions, sports medicine surgeries performed, preoperative personality traits, preoperative personality trait categorization, postoperative functional outcomes, and statistical tests used to correlate preoperative personality traits and postoperative functional outcomes. In addition to resilience, the present study has chosen to evaluate 5 additional personality traits (i.e., “grit,” “catastrophizing,” “locus of control,” “optimism,” and “self‐efficacy”), which are all closely related to a person's innate ability to overcome hardship (Table S2). Included studies were also assessed for methodologic risk of bias using the Risk of Bias in Nonrandomized Studies of Interventions tool for nonrandomized studies.^6^, ^7^ Risk‐of‐bias assessments were conducted by 2 independent reviewers, with all conflicts resolved by a third independent reviewer. Descriptive statistics were performed on all available data. Given extensive heterogeneity among procedure type, preoperative personality trait measures, and postoperative outcome measures, a meta‐analysis was not performed. Although descriptive sex and gender data were available for most studies, analyses evaluating the impact of sex and gender on outcomes were limited. Therefore, subanalyses based on sex and gender were not performed.

## RESULTS

**FIGURE 1:**
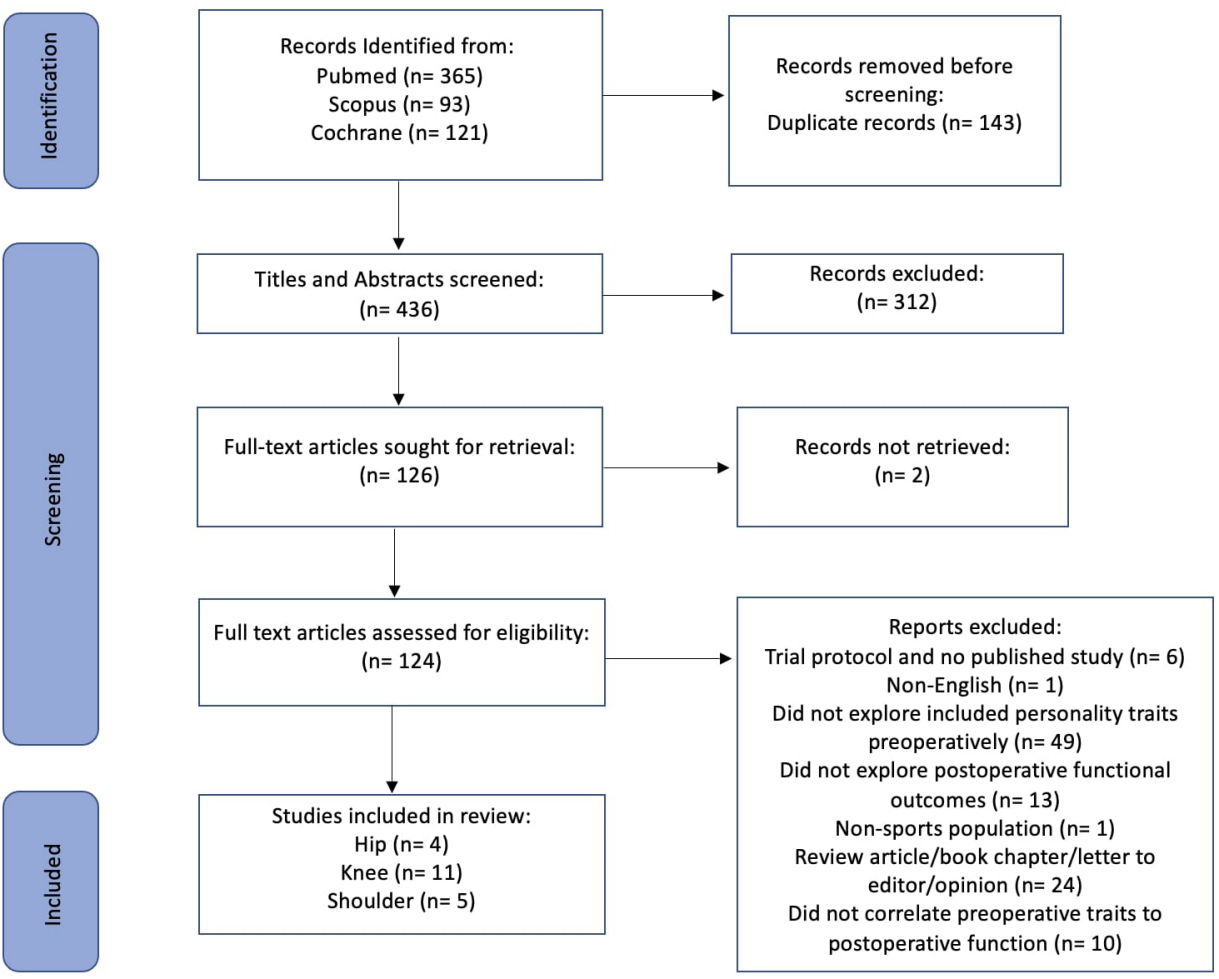
Flowchart of the article inclusion and exclusion conducted under Preferred Reporting Items for Systematic Reviews and Meta‐Analysis guidelines.

A total of 463 articles were identified from the primary search query. Following title and abstract screening, 124 articles remained and underwent full‐text review. Twenty articles met inclusion criteria and were included in this review (Figure 1).^8^, ^9^, ^10^, ^11^, ^12^, ^13^, ^14^, ^15^, ^16^, ^17^, ^18^, ^19^, ^20^, ^21^, ^22^, ^23^, ^24^, ^25^, ^26^, ^27^

Study characteristics and demographics are summarized in Table 1. A total of 1826 cases (244 hip, 560 shoulder, 1022 knee) in 1865 patients (1003 women [54%]) with an age range of 15 to 80 years were reviewed. Knee arthroscopy was the most common population (11 studies),^10^, ^17^, ^18^, ^19^, ^20^, ^21^, ^22^, ^24^, ^25^, ^26^, ^27^ followed by shoulder (5 studies)^12^, ^13^, ^14^, ^15^, ^16^ and hip (4 studies),^8^, ^9^, ^11^, ^23^ respectively. Most studies (95%) were conducted at a single institution. Study periods ranged from April 2010 to August 2022. Among included articles, there were no randomized controlled trials, 11 were prospective cohort studies (Level II evidence),^9^, ^11^, ^14^, ^15^, ^16^, ^19^, ^21^, ^22^, ^23^, ^24^, ^27^ 6 were retrospective cohort studies (Level III evidence),^10^, ^12^, ^13^, ^20^, ^25^, ^26^ 2 were prospective case series (Level III evidence),^8^, ^17^ and 1 was a case‐control study (Level II evidence).^18^ Methodologic risk‐of‐bias assessment determined that 1 study had low bias,^17^ 17 studies had moderate bias,^8^, ^9^, ^11^, ^12^, ^13^, ^14^, ^15^, ^16^, ^18^, ^19^, ^21^, ^22^, ^23^, ^24^, ^25^, ^26^, ^27^ and 2 had severe bias^10^, ^19^ (Figure S1).

**TABLE 1:** Study Characteristics and Patient Demographics of Included Studies

Author	Study Period	Study Design	Level of Evidence	Data Source	ROB/ROBINS	No. of Surgical Cases	No. of Patients (Female)	Age (Mean ± SD [Range])	Follow‐Up (Mean [Range])
*Hip*									
Clapp et al. (2020)^8^	2016 to 2017	Prospective Case Series	III	Single Institution	Moderate	85	85 (64)	33.7 ± 12.4	5.7 (5‐7) mo
Browning et al. (2021)^9^	2017 to 2019	Prospective Cohort Study	II	Single Institution	Moderate	68	68 (47)	31.9 (19‐44)	18 (12‐24) mo
Jochimsen et al. (2021)^23^	2017 to 2018	Prospective Cohort Study	II	Single Institution	Moderate	51	51 (41)	37.6 ± 12.3	3 mo
Silverman et al. (2022)^11^	NR	Prospective Cohort Study	II	Single Institution	Moderate	40	40 (17)	40 ± 15	5.7 (5‐7) mo
*Shoulder*									
Tokgoz et al. (2021)^12^	2015 to 2016	Retrospective Cohort Study	III	Single Institution	Moderate	114	114 (62)	48.5 (16‐83)	16 (12‐28) mo
Hines et al. (2022)^13^	2016 to 2019	Retrospective Cohort Study	III	Single Institution	Moderate	119	119 (48)	61 ± 10	(6‐12) mo
Wilson et al. (2022)^14^	2017 to 2020	Prospective Cohort Study	II	Single Institution	Moderate	98	98 (53)	60.8 (26‐80)	(3‐6) mo
Petrie et al. (2024)^16^	2017 to 2021	Prospective Cohort Study	II	Single Institution	Moderate	131	131 (74)	57.6 ± 9.8	3‐24) mo
Wilson et al. (2023)^15^	2017 to 2021	Prospective Cohort Study	II	Single Institution	Moderate	98	98 (50)	61.6 (26‐80)	32 (24‐47) mo
*Knee*									
Thomeé et al. (2008)^17^	NR	Prospective Case Series	III	Single Institution	Low	38	38 (13)	29.7 (16‐55)	12 mo
Ardern et al. (2013)^18^	2010 to 2011	Prospective Case Control Study	II	Single Institution	Moderate	187	187 (137)	27.3 ± 8.7	(4‐12) mo
Chavez et al. (2020)^19^	2017 to 2019	Prospective Cohort Study	II	Single Institution	Moderate	136	175 (78)	48 ± 11.5	(3‐6) mo
Drayer et al. (2020)^20^	2017	Retrospective Cohort Study	III	Single Institution	Critical	50	50 (NR)	NR	Min. of 6 mo
Everhart et al. (2020)^21^	NR	Prospective Cohort Study	II	Single Institution	Moderate	101	101 (49)	32.7	12.1 ± 7.5 mo
Hsu et al. (2020)^22^	NR	Prospective Cohort Study	II	Single Institution	Moderate	25	25 (3)	20.6 ± 4.8	12 mo
Jochimsen et al. (2020)^10^	NR	Retrospective Cohort Study	III	Multi‐ Institution	Critical	48	48 (21)	22.7 ± 4.6	(1.5‐6) mo
Armento et al. (2023)^24^	2019 to 2022	Prospective Cohort Study	II	Single Institution	Moderate	137	137 (96)	15.8 ± 2.74	(3‐12) mo
Pascual‐Leone et al. (2023)^25^	2020 to 2022	Retrospective Cohort Study	III	Single Institution	Moderate	58	58 (24)	15 ± 2.1	(0.5‐24) mo
Daniel et al. (2024)^26^	2016 to 2021	Retrospective Cohort Study	III	Single Institution	Moderate	170	170 (86)	20.1 (13‐57)	34.8 (12‐69.6) mo
Leahy et al (2024)^27^	2017 to 2022	Prospective cohort Study	II	Single Institution	Moderate	72	72 (40)	35 (16‐66)	(0‐24) mo

min, Minutes; mo, Month; NR, Normal Resilience; ROB/ROBINS, Risk Of Bias/Risk of Bias In Non‐randomised Studies; SD, Standard Deviation.

Preoperative personality measures and postoperative functional outcomes used across the included studies are summarized in Table 2. With regard to these measures, the Brief Resilience Scale (BRS), Pain Catastrophizing Scale, Knee Self‐Efficacy Score, Pain Self‐Efficacy Questionnaire, and Grit‐S have all been validated in multiple clinical settings as reliable measures of resilience, catastrophizing, self‐efficacy, and grit, respectively.^28^, ^29^, ^30^, ^31^ Details regarding personality measures are described in Table S3 and descriptions for each of the postoperative functional outcome measures can be found in Table S4. Among preoperative personality traits, resilience was most studied (9 articles), followed by pain catastrophizing (7 articles), self‐efficacy (3 articles), grit (2 articles), and locus of control (1 article), respectively (Table 2). The most common statistical tests used to correlate preoperative personality traits to postoperative functional outcomes included Spearman (4 studies) and Pearson correlation (4 studies). Six studies conducted regression analyses to determine whether preoperative personality were predictors of postoperative functional outcomes (Table 2).

**TABLE 2:** Variability in Personality Trait, Functional Outcome, and Statistical Measures Used Among Included Studies

Author	Procedure/Pathology	Preoperative Personality Measured Used	Postoperative Functional Measures Used	Statistics Used
*Hip*				
Clapp et al. (2020)^8^	FAIS	PCS	IHOT‐12, mHHS, HOOS	ROC curve analysis
Browning et al. (2021)^9^	FAIS	PCS	HOS‐SS	Bivariate correlations
Jochimsen et al. (2021)^23^	FAIS	PCS, PSEQ‐2	IHOT‐12	Multivariate logistic regressions
Silverman et al. (2022)^11^	FAIS, labral tear, internal snapping hip (both in native and artificial joints), femoral dysplasia	BRS	mHHS, HOOS	Pearson correlation
*Shoulder*				
Tokgoz et al. (2021)^12^	Labrum lesions, slap lesion, biceps pathology, subscapularis pathology, supraspinatus pathology, shoulder impingement syndrome	PCS	UCLA Shoulder Scale	Chi‐square analysis
Hines et al. (2022)^13^	Rotator cuff repair	BRS	ASES	Linear mixed model
Wilson et al. (2022)^14^	Rotator cuff repair	BRS	SANE, ASES	Spearman correlation
Petrie et al. (2024)^16^	Rotator cuff repair	BRS	SST, SANE, VR‐12P, ASES	Linear mixed effects model, Pearson correlation
Wilson et al. (2023)^15^	Rotator cuff repair	BRS	SANE, ASES	Spearman correlation
*Knee*				
Thomeé et al. (2008)^17^	ACL reconstruction	K‐SES Present, K‐SES Future	Tegner, PAS, KOOS, Muscle function	Multiple regression analyses, multiple logistic regression analyses, Spearman's rank correlation
Ardern et al. (2013)^18^	ACL reconstruction, meniscectomy, chondroplasty	SRLC, ERAIQ	Instrumented Knee Laxity, Noyes Hop Tests, IKDC, Return to sport	Binary regression
Chavez et al. (2020)^19^	Meniscectomy, chondroplasty	BRS	SANE, VR‐12, KOOS	Linear mixed model, multivariate regression
Drayer et al. (2020)^20^	Unspecified arthroscopic knee procedure	BRS	IKDC, VR‐12, KOOS, PROMIS function	Pearson correlation
Everhart et al. (2020)^21^	ACL reconstruction, meniscectomy, chondroplasty, isolated meniscus repair	PCS	Tegner, IKDC, Return to sport	Multivariate regression, Pearson correlation
Hsu et al. (2020)^22^	Meniscectomy, chondroplasty	KASE, PCS	IKDC, Muscle function	Pearson's product moment correlation
Jochimsen et al. (2020)^10^	ACL reconstruction	PCS	KOOS	Spearman correlation
Armento et al. (2023)^24^	ACL reconstruction, meniscectomy, chondroplasty, isolated meniscus repair	Grit‐S	HSS Pedi‐FABS, Neuro‐QoL, Lysholm Knee Scoring Scale, Pedi‐IKDC, PROMIS function	Linear mixed model
Pascual‐Leone et al. (2023)^25^	ACL reconstruction, meniscectomy, chondroplasty, isolated meniscus repair	5‐item Grit	Knee Extension, Knee Flexion, Range of Motion	Mann‐Whitney *U* test
Daniel et al. (2024)^26^	ACL reconstruction	BRS	Marx Activity Scale, Tegner, Lysholm Knee Scoring Scale, IKDC, VR‐12, KOOS, Range of Motion	Nominal logistic regression analyses, Cox proportional hazards model
Leahy et al (2024)^27^	ACL reconstruction	BRS	Marx Activity Scale, VR‐12	Linear mixed model

ACL, anterior cruciate ligament; ASES, American Shoulder and Elbow Surgeons; BRS, Brief Resilience Scale; ERAIQ, Emotional Responses of Athletes to Injury Questionnaire; FAIS, Femoroacetabular Impingement Syndrome Arthroscopy; HOS‐SS, Hip Outcome Score; IHOT‐12, International Hip Outcome Tool 12; IKDC, International Knee Documentation Committee; K‐SES, Knee‐Self Efficacy Scale; KASE, Knee Activity Self‐Efficacy; KOOS, Knee Injury and Osteoarthritis Outcome Score; Neuro‐QoL, Quality of Life in Neurological Disorders; PCS, Pain Catastrophizing Scale; PROMIS, Patient‐Reported Outcomes Measurement Information System; PSEQ‐2, Pain Self‐Efficacy Questionnaire‐2; Pedi‐IKDC, Pediatric International Knee Documentation Committee; ROC curve analysis, receiver‐operating characteristic curve analysis; SANE, Single Assessment Numeric Evaluation; mHHS, modified Harris Hip Score; SRLC, Sports Rehabilitation Locus of Control; SST, Simple Shoulder Test; VR‐12,Veterans RAND 12‐Item Health Survey.

**TABLE 3:** Effects of Personality Traits on Postoperative Outcomes Among Included Studies

Personality Trait	Author	Procedure	Found Preoperative Personality as a Statistically Significant Predictor/Correlator of Postoperative Functional Outcomes	Preoperative Personality Data	Outcome Summary
*Resilience*					
	Silverman et al. (2022)^11^	Hip	Yes	Low resilience group (N = 14) = BRS < 22 Normal resilience group (N = 12) = BRS between 22 and 24 High resilience group (N = 14) = BRS > 24. 2 Patients with a history of anxiety/depression (N = 7) had a mean BRS score of 20.5 (>3 points lower than patients without anxiety/depression).	Preoperative BRS scores showed a moderately positive Pearson correlation with postoperative mHHS (R = 0.36) and HOS‐Daily (R = 0.4)
	Hines et al. (2022)^13^	Shoulder	No	Mean BRS = 23.5 (N = 119)	Preoperative BRS scores were not associated with achieving a substantial clinical benefit threshold in ASES scores at 6 months postoperatively from arthroscopic rotator cuff repair.
	Wilson et al. (2022)^14^	Shoulder	No	Mean BRS = 23.5 with a range of 12‐30 (N = 98)	There was no statistically significant correlation between preoperative BRS and 3 ‐ and 6 ‐month ASES.
	Petrie et al. (2024)^16^	Shoulder	No	Low resilience group (N = 20) = BRS 3.04 ± 0.35 Normal resilience group (N = 85) = BRS 0.97 ± 0.35 High resilience group (N = 26) = BRS 4.98 ± 0.54) Overall mean (N = 131) = BRS 4 ± 0.7	Preoperative BRS scores in patients undergoing arthroscopic rotator cuff repair were significantly correlated to postoperative SST scores (R = 0.18) and VR‐12M (R = 0.31) at 12 and 24, months, respectively.
	Wilson et al. (2023)^15^	Shoulder	Yes	Mean BRS = 23.5 with a range of 12‐30 (N = 98)	Preoperative Brief Resilience Scale showed statistically significant correlations with Single Assessment Numeric Evaluation (R = 0.259).
	Chavez et al. (2020)^19^	Knee	No	Low resilience group (N = 5) Normal resilience group (N = 87) High resilience group (N = 45) Standard cutoff values for resilience were applied: 1.00‐2.99 for low resilience, 3.00‐4.30 for normal resilience, and 4.31‐5.00 for high resilience.	Preoperative BRS score did correlate with postoperative patient‐reported functional outcomes or satisfaction after knee arthroscopy.
	Drayer et al. (2020)^20^	Knee	Yes	Low resilience group (N = 9) High resilience group (N = 41). Mean preoperative and postoperative BRS were significantly different (Preoperative 27.0 HR v 18.6 LR, *P* < .001)	Active military patients with higher preoperative resilience showed markedly better early postoperative outcomes, showed by improved PROMIS‐43 (R = 0.199), KOOS (R = 0.174), and IKDC scores (R = 0.199) following sports‐related knee surgery.
	Daniel et al. (2024)^26^	Knee	Yes	Low resilience group (N = 61) BRS score = 21.1 (95% CI, 20.5‐21.7) Average resilience group (N = 42) BRS score = 24.5 (95% CI, 24.3‐24.6) High resilience group (N = 67) BRS score = 28.1 (95% CI, 27.8‐28.9) (*P* < .001).	Patients with low preoperative BRS scores exhibited worse PROMs, knee extension, and return to sport time during the early postoperative period compared with those with high and normal preoperative BRS scores.
	Leahy et al (2024)^27^	Knee	Yes	Mean BRS score of low resilience group (N = 15) = 2.9 ± 0.3 and range of 2.3‐3.2 points. Mean BRS score of normal resilience group (n = 47) = 4 ± 0.3 and a range of 3.3‐4.5 points. Mean BRS score of high resilience group (N = 10) = 5 ± 0.1 and a range of 4.7‐5.0 points. Mean BRS score overall was 3.9, ranging from 2.3 to 5.0 points.	Higher preoperative resilience scores were positively associated with better aggregated KOOS scores at 1 year. Likewise, greater resilience was associated with improved VR‐12M scores at both 1 and 2 years postoperatively. However, there were no statistically significant associations between BRS scores and achievement of minimal clinically important difference (MCID) or substantial clinical benefit (SCB).
*Catastrophizing*					
	Clapp et al. (2020)^8^	Hip	No	Mean PCS score 17.81 ± 10.13 (N = 85)	PCS scores of 25.5 as a potential predictor for achieving the MCID in functional outcomes; however, neither reached statistical significance (*P* > .05).
	Browning et al. (2021)^9^	Hip	No	Mean PCS: 17.7 ± 10.5 (N = 58)	PCS scores were not predictive of achievement of patient acceptable symptom rate (PASS) and substantial clinical benefit (SCB) on HOS‐SS.
	Jochimsen et al. (2021)^23^	Hip	Yes	Mean PCS score:19.5 ± 13.0 (N = 51)	PCS is predictive of postoperative function in hip arthroscopy population when function is gauged by iHOT‐12 score
	Tokgoz et al. (2021)^12^	Shoulder	Yes	Catastrophization group (N = 42) Noncatastrophization group (N = 42) A cutoff score of 19 was used to categorize patients with high catastrophizing Female participants reported higher levels of catastrophizing (39 out of 42, *P *= .013).	Catastrophization patient group had significantly lower functional capacity outcomes when compared with the noncatastrophization patient group
	Everhart et al. (2020)^21^	Knee	Yes	PCS subscores were reported. PCS‐catastrophizing: Mean 23.9 ± 9.6 with a range of 13‐63 PCS‐rumination: Mean 8.2 ± 3.6 with a range of 4‐20PCS‐magnification: Mean 5.7 ± 2.4 with a range of 3‐15 PCS‐helplessness: Mean 9.8 ± 4.5 with a range of 6‐29 N = 101	Severe catastrophizers, defined as PCS score > 36, were significantly more likely to be unable to return to a similar activity level after surgery (unadjusted OR 14.75 CI 1.68, 129; *P* = .002) and were significantly less likely to return to similar preinjury activity levels (OR 11.3 CI 1.51, 236; *P* = .02)
	Hsu et al. (2020)^22^	Knee	No	Mean PCS score = 11.8 with a range of 8.4‐15.2 (N = 25)	No statistically significant correlation was found between preoperative catastrophizing and postoperative functional outcomes.
	Jochimsen et al. (2020)^10^	Knee	No	PCS scores were taken at 2 time points preoperatively. The first measurement was taken within 7 days of ACL injury and the second was taken the day of surgery (DOS). Mean PCS score within 7 days of injury: 11.6 ± 10.8 with a range of 0 to 40 Mean PCS on DOS: 2.5 ± 3.7 with a range of 0 to 18 (N = 48)	Elevated pain catastrophizing was not a predictor of poor long‐term recovery.
*Self‐Efficacy*					
	Jochimsen et al. (2021)^23^	Hip	Yes	Mean PSEQ score: 38. ± 13.6 (N = 48)	Poor psychosocial health before surgery was independently linked to higher postoperative pain and diminished function, with self‐efficacy as the strongest predictor of functional outcomes in the postoperative period.
	Thomeé et al. (2008)^17^	Knee	Yes	Mean K‐SES subscores were reported. Mean K‐SES‐present score: 5.6 ± 2.3 (N = 38) Mean K‐SES‐future score: 5.9 ± 2.2 (N = 38)	Preoperative perception of self‐efficacy regarding knee function was found to reliably predict ability to regain acceptable levels of physical activity, symptom management, and muscle function up to 1 year after ACL reconstruction.
	Hsu et al. (2020)^30^	Knee	Yes	Mean KASE = 56 range of 47.8 to 64.3 (N = 25)	Higher presurgery Knee Activity Self Efficacy (KASE) score was associated with greater quadriceps peak torque at 1 year after surgery (r = 0.48, *P *= .02).
*Grit*					
	Armento et al. (2023)^24^	Knee	Yes	Mean 5‐item Grit score was 3.70 ± 0.54 (N = 137)	Baseline grit showed statistically significant correlations with HSS Pedi‐FABS and NeuroQoL lower extremity scores. However, no significant associations were observed between baseline grit and IKDC, Lysholm, or PROMIS pain scores or mobility outcomes.
	Pascual‐Leone et al. (2023)^25^	Knee	Yes	Mean Grit scale score = 4.2 ± 1.0 (N = 58)	Patients with preoperative grit scores below the 50th percentile showed 5 degrees less total range of motion (ROM) at 3 months after surgery compared with those with higher grit scores following meniscus repair.
*Locus of Control*					
	Ardern et al. (2013)^18^	Knee	No	Participants cohorts were created based on whether participants were able to return to preinjury levels of sport. SRLC scores were reported as subscores. ERAIQ score of those who returned to sport = 54.2 ± 24.7 ERAIQ score of those who did not return to sport = 56.2 ± 22.8 SRLC‐Internal score of those who returned to sport = 4.3 ± 1.7 SRLC‐Internal score of those who did not return to sport = 4.6 ± 1.7 SRLC‐Powerful Others score of those who returned to sport = 8.8 ± 2.1 SRLC‐Powerful Others score of those who did not return to sport = 8.8 ± 2.2 SRLC‐Chance score of those who returned to sport = 11.9 ± 2.0 SRLC‐Chance score of those who did not return to sport = 11.6 ± 2.4	Locus of control both preoperatively and during early recovery showed no statistically significant association with returning to preinjury sports levels at 1 year.

ACL, anterior cruciate ligament; ASES, American Shoulder and Elbow Surgeons; BRS, Brief Resilience Scale; CI, confidence interval; ERAIQ, Emotional Responses of Athletes to Injury Questionnaire; HOS‐SS, Hip Outcome Score; HR, high resilience; HSS Pedi FABS, Hospital for Special Surgery Pediatric Functional Activity Brief Scale; IHOT‐12, International Hip Outcome Tool 12; IKDC, International Knee Documentation Committee; K‐SES, Knee‐Self Efficacy Scale; KASE, Knee Activity Self‐Efficacy; KOOS, Knee Injury and Osteoarthritis Outcome Score; KT‐1000, Knee Tester‐1000; LR, low resilience; MCS, mental component score; mHHS, modified Harris Hip Score; NR, normal resilience; OR, odds ratio; PCS, Pain Catastrophizing Scale; PROMIS, Patient‐Reported Outcomes Measurement Information System; PSEQ, Pain Self‐Efficacy Questionnaire; ROM, range of motion; SD, standard deviation; SRLC, Sports Rehabilitation Locus of Control; SST, Simple Shoulder Test; VAS, visual analog scale; VR 12, Veterans Rand 12.

### Resilience

Table 3 details findings from the 9 studies that investigated resilience. All studies used the BRS to measure preoperative resilience. Of note, there is currently no available literature that has identified a minimal clinically important difference for the BRS. More than half of the studies investigating resilience found an association (5/9, 56%), compared with 4 studies that did not (4/9, 46%). Five studies (1 hip, 1 shoulder, and 3 knee) found that high preoperative resilience when measured by the BRS was an indicator of statistically superior postoperative functional outcomes. The functional tests found to have a statistically significant correlation with the BRS were the modified Harris Hip Score, Patient‐Reported Outcomes Measurement Information System, International Knee Documentation Committee Questionnaire, and Lysholm Knee Scoring Scale. The remaining 4 studies (3 shoulder and 1 knee) found no statistically significant correlation between preoperative BRS and postoperative functional measures.

### Pain Catastrophizing

Table 3 details findings from the 7 studies that explored pain catastrophizing. The Pain Catastrophizing Scale was used in all 7 included studies to measure preoperative pain catastrophizing. The majority of studies (2 hip and 2 knee—4/7, 57%) evaluating pain catastrophizing did not find a statistically significant correlation with postoperative outcomes. The remaining 3 studies (1 knee, 1 hip, 1 shoulder, 43%) found a statistically significant relationship between preoperative pain catastrophizing and postoperative outcomes.

### Self‐Efficacy

Table 3 details the findings of the 3 studies (1 hip and 2 knee) that investigated self‐efficacy. Each study used different measures for preoperative self‐efficacy (i.e., Knee Self Efficacy Score, Knee Activity Self‐Efficacy, Pain Self‐Efficacy Questionnaire) (Table 3). All studies evaluating self‐efficacy found a statistically significant association between preoperative self‐efficacy and the postoperative functional measures. The single hip study found a significant relationship between preoperative Pain Self‐Efficacy Questionnaire score and International Hip Outcome Tool 12 postoperatively.^23^ Among the 2 knee studies, higher preoperative self‐efficacy scores were significantly correlated with improved postoperative Tegner, Knee Injury and Osteoarthritis Outcome Score, and peak quadriceps strength.^17^, ^22^

### Grit

Table 3 details the findings of the 2 studies that explored preoperative grit. Pascual‐Leone et al. found a statistically significant correlation across preoperative 5‐item Grit scale scores and postoperative knee extension, knee flexion, and range of motion. Similarly, Armento et al. identified a statistically significant correlation between preoperative Grit scale and postoperative Hospital for Special Surgery Pediatric Functional Activity Brief Scale and Quality of Life in Neurological Disorders scores. However, preoperative Grit scale scores did not have predictive ability for other functional tests of the knee, including International Knee Documentation Committee or Lysholm.

### Locus of Control and Optimism

A single study investigated locus of control and optimism in recreational‐ and competitive‐level athletes using the Sports Rehabilitation Locus of Control and Emotional Responses of Athletes to Injury Questionnaire, respectively.^18^ No statistically significant correlation existed between locus of control or optimism and postoperative clinical or return‐to‐sport measures.

## DISCUSSION

This study found that most studies investigating resilience, self‐efficacy, and grit found statistically significant positive correlations between these traits and postoperative functional outcomes. However, in the remaining studies analyzing pain catastrophizing, locus of control, and optimism, there was a combination of mixed findings or no correlation with outcomes. By understanding the impact of these relationships, this review may provide actionable data for the development of targeted cognitive or behavioral interventions to optimize patients undergoing orthopaedic sports medicine surgery.

### Resilience

The present review found that high preoperative resilience was associated with better postoperative functional outcomes in 5 (1 study on intra‐articular hip pathology, 1 study on rotator cuff repair, and 3 studies on knee arthroscopy) out of 9 studies. In particular, preoperative resilience was found to be an effective predictor of clinical outcomes in the majority of published knee arthroscopy cohorts. These findings suggest that baseline resilience, as a personality trait, has the potential to play a role in the postoperative course. Importantly, however, it should be acknowledged that more extensive injuries and subsequent procedures/postoperative course (e.g., anterior cruciate ligament reconstruction plus extended rehabilitation vs meniscectomy) may influence a patient's preoperative outlook and resilience scores. Clinically, this implies that incorporating resilience‐focused strategies prior to knee surgery may enhance functional outcomes, patient satisfaction, and mental well‐being postoperatively.

Four studies found no correlation between resilience and postoperative outcomes.^13^, ^14^, ^16^, ^19^ Among these, Chavez et al.^32^ explored resilience in knee meniscectomy/chondroplasty patients and the 3 remaining studies investigated shoulder arthroscopy cohorts.^13^, ^14^, ^16^ Among the 3 shoulder studies, primary outcomes of American Shoulder and Elbow Surgeons scores did not correlate with BRS. Although current literature suggests limited impact of resilience on shoulder arthroscopy outcomes, further high‐level studies are warranted. Moreover, with respect to all cohort, future research should also consider alternate for resilience such as the Connor‐Davidson Resilience Scale, which has been shown to be predictive of functional outcomes in shoulder arthroplasty.^33^ In comparison with the BRS, which more narrowly evaluates an individual's ability to rebound following challenges, the Connor‐Davidson Resilience Scale more broadly measures resilience including external resources and capacities that facilitate adaptation to stress or adversity.^34^

### Pain Catastrophizing

The present review noted mixed findings regarding pain catastrophizing, with 4 of 7 studies reporting no significant correlation between preoperative catastrophizing and postoperative outcomes. Pain catastrophizing is a potentially modifiable personality trait that may be optimized to improve postoperative results. For instance, interventions such as Acceptance and Commitment Therapy have been shown to significantly alleviate pain perception in individuals with chronic pain.^35^ There are data to suggest that pain interference and physical function show poor longitudinal association in people living with chronic pain and should therefore be viewed as separate constructs.^36^ Although pain catastrophizing amplifies the perception of pain and emotional distress, it may not significantly impact functional recovery. Some patients with high levels of pain catastrophizing may still achieve functional improvements through structured rehabilitation, despite reporting persistent pain interference. Conversely, others may experience pain relief postoperatively due to the anatomical disorder being addressed but remain functionally limited due to fear of movement or avoidance behaviors rooted in catastrophizing tendencies.

### Self‐Efficacy

All 3 studies examining self‐efficacy found significant associations between high preoperative self‐efficacy and better postoperative functional outcomes as measured by the International Hip Outcome Tool 12, Tegner, Knee Injury and Osteoarthritis Outcome Score, and muscle function. This underscores the role of self‐efficacy in optimizing recovery, suggesting that patients with greater confidence in their ability to manage postoperative challenges generally fare better. Previous systematic reviews have observed that self‐efficacy positively influences compliance with discharge instructions and locomotion recovery after acute injuries.^37^ Self‐efficacy positively affects compliance with discharge instructions, which can help reduce the likelihood of adverse events and surgical complications. Additionally, it may also offer a pathway that health care providers can take to predict which patients may fare better with the proposed surgical treatment plan. Identifying patients with low self‐efficacy preoperatively can facilitate targeted interventions aimed at optimizing these traits. Techniques such as imagery rescripting, goal‐setting interventions, and motivational interviewing have been shown to boost self‐efficacy.^38^, ^39^

### Grit, Locus of Control, and Optimism

Previous research has identified an association between grit and treatment adherence as well as the likelihood of following postoperative rehabilitation protocols.^40^ The included studies on grit yielded mixed results. It should be noted that only 2 studies that investigated grit met the inclusion criteria. As such, limited data limited the strength of the present findings. The absence of a statistically significant trend may be attributed to differences in patient populations and the variations in the personality trait measures used across studies (e.g. Grit scale vs 5‐item Grit). Grit, characterized by perseverance and passion for long‐term goals, could be nurtured through interventions such as perseverance training and long‐term goal setting, which may, in turn, enhance postoperative recovery. The single study on locus of control and optimism found no significant association with postoperative outcomes in patients who underwent knee surgeries.^18^ Ardern et al.,^18^ who used return to sport as a functional measure, recorded the Sports Rehabilitation Locus of Control and Emotional Responses of Athletes to Injury Questionnaire to assess locus of control and optimism, respectively. As only 1 study on this trait has been conducted to date, future research should be performed across different sports medicine populations (e.g., knee, hip, and shoulder) to reliably ascertain whether grit and locus of control are indeed personality traits that can predict a patient's postoperative outcome.

### Limitations

This study has several limitations. First, this study focused exclusively on preoperative personality traits and their association with postoperative function and therefore does not account for the dynamic nature of personality traits, which can evolve postoperatively. Another limitation is the exclusion of certain traits due to the study design. The choice to selectively analyze specific personality traits—namely, resilience, catastrophizing, self‐efficacy, grit, locus of control, and optimism—led to the exclusion of other potentially relevant traits (i.e., perseverance, self‐esteem, neuroticism, helplessness, etc.). Third, some personality trait cohorts (e.g., grit) were represented by only a few studies, making it challenging to draw definitive conclusions regarding their predictive ability. Additionally, the reliance on a single measure to assess a specific preoperative personality trait in many studies limited the robustness of the analysis. For example, almost all studies used the Pain Catastrophizing Scale to assess pain catastrophizing and BRS to assess resilience. Finally, it is important to acknowledge that the conclusions drawn from this review must be interpreted within the context of each included study. Variability in study design, patient populations, and outcome measures limits the ability to make generalized conclusions.

## CONCLUSIONS

Resilience, self‐efficacy, and grit emerged as personality traits with potential associations with postoperative functional outcomes in orthopaedic sports medicine patients. The remaining personality traits of pain catastrophizing, locus of control, and optimism showed either mixed results or no correlation with functional outcomes. When assessed, no study found a correlation between preoperative traits and achievement of minimal clinically important difference in outcomes.

## SUPPORTING INFORMATION

Additional supporting information can be found online in the Supporting Information section.

## DISCLOSURES

The authors (C.J.R., T.L.T., H.S.F., M.M., G.S.G., F.P.T., K.B.F.) declare that they have no known competing financial interests or personal relationships that could have appeared to influence the work reported in this paper.

## Supporting information

Supplementary Material
