# Supplementary material for: Preoperative Resilience, Self‐Efficacy, and Grit Are Associated With Postoperative Functional Outcomes in the Sports Medicine Patient: A Systematic Review
Source: Arthrosc Sports Med Rehabil. 2026 May 21:e70031. Online ahead of print. doi: 10.1002/ars2.70031 (PMC13399892; doi:10.1002/ars2.70031)
Supplement: Supplementary file 1 — Supplementary Material [file ARS2-9999-e70031-s001.pdf]

Supplementary Table 1. Database Search Strategy

Database	PubMed (MEDLINE), Scopus (EMBASE, MEDLINE, COMPENDEX)
**Date**	October 1, 2024
**Strategy**	Population AND Outcome
**Population**	anterior cruciate ligament repair OR ACL repair OR ACL-repair OR anterior cruciate ligament reconstruction OR ACL reconstruction OR ACL-reconstruction OR meniscectomy OR meniscus repair OR distal biceps reconstruction OR distal biceps repair OR elbow arthroscopy OR lateral collateral ligament repair OR LCL repair OR LCL-repair OR LCL reconstruction OR LCL-reconstruction OR ulnar collateral ligament reconstruction OR UCL reconstruction OR UCL-reconstruction OR Achilles tendon repair OR Achilles tendon reconstruction OR hip arthroscopy OR hip capsule reconstruction OR labral reconstruction OR labral repair OR labrum reconstruction OR labrum repair OR quadriceps tendon repair OR quadriceps tendon reconstruction OR posterolateral corner repair OR posterolateral corner reconstruction OR PCL repair OR PCL-repair OR PCL-reconstruction OR PCL reconstruction OR posterior cruciate ligament repair OR PCL repair OR PCL-repair OR PCL reconstruction OR PCL-reconstruction OR posterior cruciate ligament reconstruction OR patellar tendon repair OR patellar tendon reconstruction OR bankhart repair OR bankhart reconstruction OR biceps tenotomy OR biceps tenodesis OR rotator cuff repair OR shoulder arthroscopy OR sternoclavicular joint OR subacromial decompression OR capsular reconstruction OR capsular repair OR ankle arthroscopy OR knee arthroscopy
**Intervention**	resilience OR psychological resilience OR resiliences OR resiliency OR resiliencies OR psychological resilience OR psychological resiliency OR stress immunity OR stress immunities OR resilient response OR resilience response OR psychological resilience response OR psychological resilient response OR brief resilience score OR brief resilience scale OR Minnesota Multiphasic Personality Inventory OR Millon Clinical Multiaxial Inventory OR connor-davidson resilience scale OR connor-davidson resilience score OR connor davidson resilience scale OR connor davidson resilience score OR life orientation test OR self-efficacy OR self efficacy OR locus of control OR catastrophe OR catastrophizing OR grit
**Outcome**	outcomes OR outcome OR outcome measures OR outcome measure OR pain OR function OR functional OR independence OR quality of life OR quality-of-life OR return to sport OR return to work OR return to activity OR return-to-sport OR return-to-work OR return-to-activity OR satisfaction OR patient satisfaction OR functional outcome OR functional outcomes OR PROM OR PROMs OR patient reported outcome measure OR patient reported outcome measures OR patient-reported outcome measures OR patient-reported outcome measure

Supplementary Table 2. Personality Trait Definitions

Personality Trait	Definition
Resilience	The process and outcome of successfully adapting to difficult or challenging life experiences, especially through mental, emotional, and behavioral flexibility and adjustment to external and internal demands
Grit	Firmness of mind or spirit: unyielding courage in the face of hardship
Catastrophizing	To imagine the worst possible outcome of an action or event: to think about a situation or event as being a or having a potentially catastrophic outcome
Self-efficacy	Belief in one's capacities to organize and execute the course of action required to produce given attainments
Locus of control	Belief about whether life events are due to own actions (internal) or due to outside forces beyond your control (external)

Supplementary Table 3. Descriptions of Preoperative Personality Trait Measures Utilized

**Preoperative Personality Trait Measures**	**Description**
Brief Resilience Scale (BRS)	The BRS is a researched tool that measures a patient's resiliency after trauma or stress
Pain Catastrophizing Scale (PCS)	The Pain Catastrophizing Scale (PCS) is a self-report tool designed to evaluate the tendency to catastrophize pain among both clinical and nonclinical groups. Comprising 13 statements, the PCS captures various thoughts and emotions individuals may encounter when experiencing pain. The statements are categorized into three dimensions: rumination, magnification, and helplessness, with responses rated on a 5-point Likert scale.
Pain Self-Efficacy Questionnaire-2 (PSEQ-2)	The Pain Self-Efficacy Questionnaire-2 (PSEQ-2) is a brief, two-item adaptation of the original Pain Self-Efficacy Questionnaire (PSEQ) that assesses an individual's confidence in their capacity to engage in activities and meet personal goals despite experiencing pain. This tool is applicable in both clinical and research contexts, providing insight into a person's resilience and coping mechanisms during painful conditions. Additionally, it aids in creating a framework to understand the psychological factors that affect an individual's reaction to injury or discomfort.
Knee-Self Efficacy Scale (K-SES)	The Knee-Self Efficacy Scale (K-SES) is a self-report instrument designed to evaluate individuals' perceived self-efficacy regarding knee function following an ACL injury. Research has demonstrated that the K-SES possesses strong reliability, validity, and responsiveness, making it a valuable tool for assessing self-efficacy during the rehabilitation process.
Sports Rehabilitation Locus of Control (SRLC)	The Sports Rehabilitation Locus of Control (SRLC) scale was created to examine the behaviors of athletes as they recover from injuries. Tailored specifically for this purpose, the SRLC adapts certain items from the Multidimensional Health Locus of Control (MHLC) questionnaire to effectively assess locus of control among injured athletes.
Emotional Responses of Athletes to Injury Questionnaire (ERAIQ)	The Emotional Responses of Athletes to Injury Questionnaire (ERAIQ) assesses athletes' emotional reactions to injuries and gauges their anxieties regarding a return to sports. This tool can be employed during the initial evaluation to inform the development of psychologically-based interventions, aiding athletes in managing their injuries effectively.
Knee Activity Self-Efficacy (KASE)	The Knee Activity Self-Efficacy (KASE) is a 10-item questionnaire designed to measure confidence in performing functional activities related to the knee. Developed by the authors from an existing questionnaire, it includes statements such as, "I can hop on the injured leg," which respondents rate on a scale from 1 ("strongly disagree") to 10 ("strongly agree"). Total scores can range from 0 to 100, with higher scores reflecting greater self-efficacy in knee-related activities.
Grit Scale	The Grit Scale is an 8-item questionnaire that serves as a more streamlined assessment of grit, adapted from the original 12-item version and validated for use with adolescents. Participants rate the extent to which various statements reflect their characteristics on a 5-point Likert scale, ranging from "not like me at all" to "very much like me."
5-Item Grit	The 5-item Grit Scale is a self-assessment tool designed to evaluate an individual's grit, defined as the capacity to pursue long-term objectives with both passion and determination. Comprising 10 statements, respondents indicate their level of agreement with each statement by selecting a number from 1 (Very much like me) to 5 (Not at all like me).

Supplementary Table 4. Descriptions of Postoperative Outcome Measures Utilized

**Postoperative Outcome Measure**	**Description**
International Hip Outcome Tool 12 (iHOT-12)	The International Hip Outcome Tool 12 (iHOT-12) is a questionnaire that measures a patient's health-related quality of life after hip disorder treatment. It's a shorter version of the original iHOT-33, which has 33 items, and is often used in clinical practice because it's easier to administer. The iHOT-12 is divided into four sections, or factors, and includes 12 questions about the patient's symptoms, limitations, and concerns:<br>Factor 1: Symptoms and functional limitations (items 1, 2, 3, 4)<br>Factor 2: Sports and recreational activities (items 6, 7, 11)<br>Factor 3: Job-related concerns (item 5)<br>Factor 4: Social, emotional, and lifestyle (items 8, 9, 10, 12)

Modified Harris Hip Score (mHHS)	The Modified Harris Hip Score (mHHS) is a specialized questionnaire aimed at evaluating a patient's functional capacity related to hip health, encompassing aspects such as pain, gait, and the ability to perform daily activities. This measure derives from the original Harris Hip Score (HHS) but focuses exclusively on the patient-reported elements. Additionally, the mHHS incorporates certain modifications within the pain and functional ability domains to enhance its relevance and accuracy.
Hip Disability and Osteoarthritis Outcome Score (HOOS)	The Hip Disability and Osteoarthritis Outcome Score (HOOS) is a comprehensive questionnaire designed to evaluate a patient's hip health and associated issues. It is utilized to assess symptoms and functional limitations in individuals undergoing therapy for hip disabilities, whether or not they have hip osteoarthritis (OA).
University of California, Los Angeles (UCLA) Shoulder Score	The University of California, Los Angeles (UCLA) Shoulder Score is a survey used to evaluate shoulder conditions and the functional outcomes following treatment. This assessment includes a mix of subjective and objective questions answered by both the patient and the physician.
Veterans RAND 12-Item Health Survey (VR-12)	The Veterans RAND 12-Item Health Survey (VR-12) is a self-administered questionnaire that measures a patient's health-related quality of life. The survey consists of 12 questions that relate to eight physical and mental health domains, including:<br>General health perceptions<br>Physical functioning<br>Role limitations due to physical problems<br>Role limitations due to emotional problems<br>Bodily pain<br>Energy fatigue<br>Social functioning<br>Mental health
American Shoulder and Elbow Surgeons (ASES)	The American Shoulder and Elbow Surgeons (ASES) score is a 100-point assessment that measures two key aspects of shoulder function: pain and the ability to perform daily activities. Each of these two dimensions contributes 50 points to the overall score.
Single Assessment Numeric Evaluation (SANE)	The Single Assessment Numeric Evaluation (SANE) is a one-item patient-reported outcome measure (PROM) that allows individuals to assess their functional status by rating how normal they feel regarding a particular joint or issue.
Simple Shoulder Test (SST)	The Simple Shoulder Test (SST) is a self-administered questionnaire designed to evaluate the functional impairments associated with shoulder dysfunction. Comprising 12 questions that require a yes or no answer, the SST examines the shoulder's capacity to perform various activities, such as dressing, bathing, lifting, carrying, and throwing.
International Knee Documentation Committee (IKDC)	International Knee Documentation Committee survey is a patient reported outcome measure that assess knee related symptoms, function and sports activity.
Pediatric International Knee Documentation Committee (Pedi-IKDC)	The Pediatric International Knee Documentation Committee (Pedi-IKDC) is a questionnaire that measures symptoms, function, and sports activity in children and adolescents with knee disorders
Patient Reported Outcomes Measurement Information System (PROMIS)	PROMIS® (Patient Reported Outcomes Measurement Information System) is a comprehensive system that offers reliable and precise assessments of patient-reported health status, encompassing various dimensions of well-being, including physical, mental, and social health.
Tegner	The Tegner Activity Scale (TAS) is a standardized tool designed to assess a patient's activity level both prior to and following an injury. It can also be utilized to evaluate participation in sports and work-related activities. The scale ranges from 0 to 10, where 10 indicates the highest level of activity and 0 represents the lowest.
Marx Activity Scale	The Marx Scale is comprised of four questions that focus on activities such as running, cutting, decelerating, and pivoting. Respondents are requested to indicate how often they engaged in each activity during their healthiest state over the previous year.
Physical Activity Scale (PAS)	The Physical Activity Scale (PAS) is a self-assessment tool that allows individuals to rate their level of physical activity on a scale from 1 to 4. A score of "1" signifies no physical activity, while "4" represents vigorous, strenuous activity. Participants use this scale to subjectively evaluate the intensity and frequency of their exercise routines.

Knee Injury and Osteoarthritis Outcome Score (KOOS)	The Knee Injury and Osteoarthritis Outcome Score (KOOS) is a specialized tool designed to capture patients' perceptions regarding their knee and related issues. It assesses both the immediate and long-term effects of knee injuries, providing insights into the patient's overall condition.
Hospital for Special Surgery Pediatric Functional Activity Brief Scale (HSS Pedi-FABS)	The Hospital for Special Surgery Pediatric Functional Activity Brief Scale is a tool that consists of eight straightforward questions aimed at evaluating the frequency with which a child engages in basic athletic movements, such as running, decelerating, and pivoting, over the course of a week. It is intended to be completed directly by young patients aged 10 to 18, although parents can assist if necessary.
Quality of Life in Neurological Disorders (Neuro-QoL™)	Neuro-QoL™ (Quality of Life in Neurological Disorders) is a measurement system designed to assess and track the physical, mental, and social impacts faced by both adults and children affected by neurological disorders.
Lysholm Knee Scoring Scale	The Lysholm Knee Scoring Scale is a 100-point questionnaire used to evaluate a patient's knee symptoms and functional abilities. This patient-reported tool features subscales that cover aspects such as pain, instability, locking, swelling, limping, stair climbing, squatting, and the requirement for support.
Hip Outcome Score (HOS)	The Hip Outcome Score (HOS) is a patient-reported outcome measure created to evaluate the effectiveness of treatment interventions, specifically arthroscopic hip surgery, in individuals with acetabular labral tears. This self-report instrument was designed to assess the results of such surgical procedures.
Knee Extension	Knee extension is the straightening of the knee joint, which increases the angle between the thigh and lower leg. Most individuals have around 0 degrees of knee extension (a fully straightened knee) and 135 degrees of knee flexion.
Knee Flexion	Knee flexion is the action of decreasing the angle between the thigh and lower leg, essentially bending the knee. Typically, a healthy knee can flex between 120 to 140 degrees.
Range of Motion	A knee range of motion (ROM) test assesses the knee's ability to move freely. It can include active and passive movements, and can evaluate extension, flexion, and rotation.

SUPPLEMENTARY FIGURE 1. Risk of bias assessment

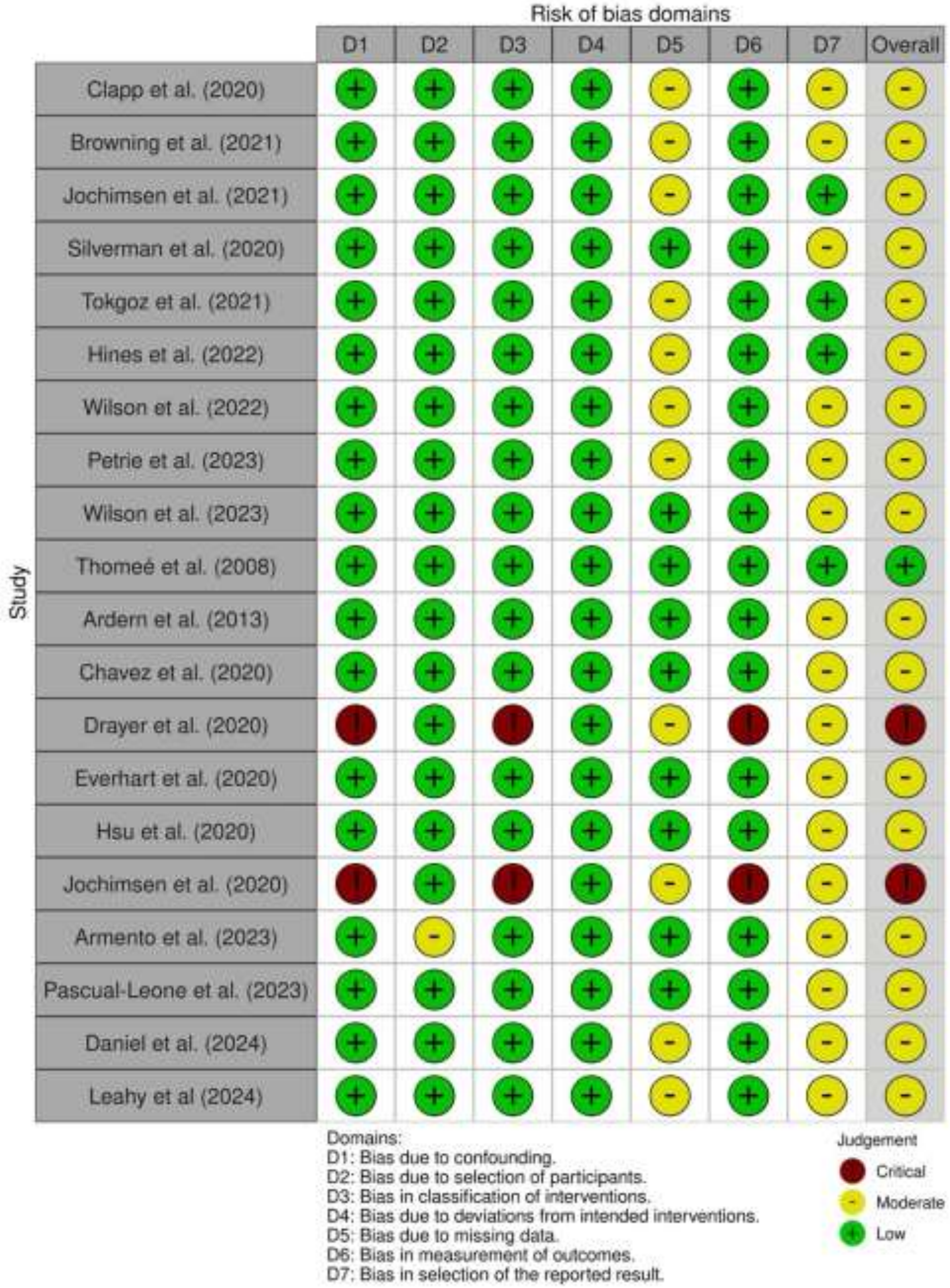
Risk of bias domains
D1
D2
D3
D4
D5
D6
D7
Overall
Study
Clapp et al. (2020)
Browning et al. (2021)
Jochimsen et al. (2021)
Silverman et al. (2020)
Tokgoz et al. (2021)
Hines et al. (2022)
Wilson et al. (2022)
Petrie et al. (2023)
Wilson et al. (2023)
Thomeé et al. (2008)
Ardern et al. (2013)
Chavez et al. (2020)
Drayer et al. (2020)
Everhart et al. (2020)
Hsu et al. (2020)
Jochimsen et al. (2020)
Armento et al. (2023)
Pascual-Leone et al. (2023)
Daniel et al. (2024)
Leahy et al (2024)
Domains:
D1: Bias due to confounding.
D2: Bias due to selection of participants.
D3: Bias in classification of interventions.
D4: Bias due to deviations from intended interventions.
D5: Bias due to missing data.
D6: Bias in measurement of outcomes.
D7: Bias in selection of the reported result.
Judgement
Critical
Moderate
Low
